# 24-Hour Physical Behavior Balance for Better Health for All: “The Sweet-Spot Hypothesis”

**DOI:** 10.1186/s40798-021-00394-8

**Published:** 2021-12-20

**Authors:** Andreas Holtermann, Charlotte Lund Rasmussen, David M. Hallman, Ding Ding, Dorothea Dumuid, Nidhi Gupta

**Affiliations:** 1 National Research Centre for the Working Environment, Copenhagen, Denmark; 2 Department of Sports Science and Clinical Biomechanics, University of Southern Denmark, Odense, Denmark; 3 Centre for Musculoskeletal Research, Department of Occupational Health Sciences and Psychology, University of Gävle, Gävle, Sweden; 4 Prevention Research Collaboration, Sydney School of Public Health, Faculty of Medicine and Health, The University of Sydney, Camperdown, NSW Australia; 5 Charles Pekins Centre, The University of Sydney, Camperdown, NSW Australia; 6 Alliance for Research in Exercise, Nutrition and Activity, Allied Health and Human Performance, University of South Australia, Adelaide, SA Australia

**Keywords:** Physical activity, Sedentary behavior, Social health inequalities, Occupational health

## Abstract

“Sit less–move more” has been the univocal advice to adults for better health. Predominantly, this advice is based on research of physical behaviors during leisure-time. A recent study among > 100,000 adults indicates a u-shaped association between leisure-time physical activity and risk for cardiovascular disease and mortality among adults in physically active occupations. This may be explained by the considerable difference in 24-h physical behaviors between adults in sedentary and physically active occupations. Thus, the advice “sit less–move more” might not be the best for health among adults in physically active occupations. To provide a scientific approach and encourage research on 24-h physical behaviors and health for those in physically active occupations, we propose the “Sweet-Spot Hypothesis.” The hypothesis postulates that the “Sweet-Spot” of 24-h physical behaviors for better health differs between adults, depending on their occupation. Specifically, the hypothesis claims that the advice “sit less–move more” does not bring adults in physically active occupations toward their “Sweet-Spot” of 24-h physical behaviors for better health. The purpose of our paper is to encourage researchers to test this proposed hypothesis by describing its origin, its theoretical underpinning, approaches to test it, and practical implications. To promote health for all, and decrease social health inequalities, we see a great need for empirically testing the “Sweet-Spot Hypothesis.” We propose the “Sweet-Spot Hypothesis” to encourage discussion, debates, and empirical research to expand our collective knowledge about the healthy “24-h physical behavior balance” for all.

## Key Points

- The univocal advice “sit less–move more” might not be the best for health for all adults.
- We propose the “Sweet-Spot Hypothesis” postulating that the “healthy balance” of 24-h physical behaviors differs between adults in sedentary and physically active occupations.
- Our main aim is to suggest a new scientific approach and to encourage research on healthy 24-h physical behavior balance for all.

## Background

The benefits of moderate-to-vigorous physical activity (MVPA) in preventing and managing a range of diseases and conditions are well established [1, 2]. Meanwhile, evidence suggests that spending prolonged sedentary hours impairs health [3]. Thus, globally “sit less–move more” has become the univocal advice for achieving better health among the general adult population [1, 2, 4].

Another recommendation conveyed from the recent WHO Guidelines on physical activity and sedentary behavior is that MVPA attenuates the detrimental health effects from many sedentary hours spent per day [1]. This is supported by a meta-analysis of accelerometer-based cohorts, finding that as little as 30 min daily MVPA attenuates the increased risk of all-cause mortality from several hours of daily sedentary behavior [5].

Emerging evidence on the joint effects of multiple physical behaviors, such as that reviewed by the WHO Guidelines on physical activity and sedentary behaviors [1] and the Canadian 24-h movement guidelines [4], recommends a new “balanced approach” to multiple behaviors and risk factors. This approach suggests that some physical behaviors (e.g., MVPA) restore “a balance” among adults spending too much time in unhealthy physical behaviors (e.g., excessive sedentary behavior) [1, 4]. For example, this new recommendation presents a range of options for sedentary populations to attenuate their higher risk for impaired health by reducing their sedentary time, increasing MVPA, or a combination of both [1, 4]. The premise of the 24-h movement guidelines is that there are “healthy” daily durations of physical behaviors. For example, the Canadian 24-h guidelines for adults propose that a healthy day includes 7–9 h sleep, 8 h or less sedentary time (with < 3 h screen time), several hours of light physical activity and at least ~ 20 min MVPA (150 min/week) [4]. The guidelines are accompanied by three core recommendations: “move more,” “reduce sedentary time” and “get sufficient sleep.”

The advantage of 24-h guidelines is that they incorporate all daily physical behaviors. Unlike single-behavior guidelines, 24-h guidelines acknowledge that physical behaviors are intrinsically linked. Thus, guidelines impacting one behavior will inevitably impact the other behaviors. The 24-h guidelines also provide flexibility in how they can be achieved, meaning they are broadly applicable. However, the core recommendations for physical activity and sedentary time which accompany the guidelines, (i.e., “sit less” and “move more”) appear more suited to the large proportion of adults who spend many hours sedentary each day (e.g., office workers). For this population, “moving more” and “sitting less” after work could bring their 24-h physical behaviors closer toward a “healthy balance” and compliant with the 24-h guidelines. However, adults in physically active occupations may already be compliant with the 24-h guidelines, provided they are getting recommended amounts of sleep. Current 24-h guidelines appear to have nothing further to offer this population of adults, although their mandatory high levels of physical activity may not represent a healthy daily movement behavior balance. Because the guidelines have no upper limit for MVPA, nor lower limit for sedentary time, these adults would be encouraged to “move more” and “sit less.” Accordingly, adults in physically active occupations could potentially be encouraged to move away from a “healthy balance,” which may lead to chronic strains, injury or fatigue. Adults in physically active occupations comprise a considerable proportion of the working population in low- and middle-income countries (LMIC), as well as in high-income countries [6, 7]. Eurofound reported that 32% of workers in the EU have a physically active occupation in terms of carrying or moving heavy loads (i.e., at least ¼ of the working day) [8]. This was almost doubled (59%) in low-skilled manual occupations, where 39% reported to rarely or never be able to take a break when they wish, and 54% reported to never sit at work. For the many adults whose primary source of physical activity is work [7], advice like “sit less–move more” may not be conducive for promoting a “healthy balance” of physical behaviors. Moreover, this group of adults might require more sleep to recover from their physically demanding workdays than adults with sedentary jobs.

A recent study among > 100,000 adults indicated a u-shaped association between leisure-time physical activity and the risk for cardiovascular disease and mortality among adults in physically active occupations [9]. This study indicates that the “healthy balance” of 24-h physical behaviors is not necessarily achieved by adhering to the advice “sit less–move more” among adults in physically active occupations. Accordingly, the WHO physical activity and sedentary behavior guidelines development group encouraged research on domain-specific physical behaviors and health [10]. They stated that “the optimal balance between occupational activity and sedentary behavior over the course of the workday” remains to be established [10]. Here, we frame this “optimal balance” of 24-h physical behaviors for better health the “Sweet-Spot” of 24-h physical behaviors (i.e., sedentary behavior, active behaviors and sleep).

We hypothesize that the “Sweet-Spot” of 24-h physical behaviors for better health differs between adults in sedentary and physically active occupations. Specifically, our hypothesis suggests that the advice “sit less–move more” may not bring adults in physically active occupations toward their “Sweet-Spot” of 24-h physical behaviors for better health.

It is well established that physically active occupations often, although not exclusively, relate to lower socioeconomic position, including lower income, occupational class and education [6, 11]. Conversely, health-promoting physical activity during leisure-time is associated with higher socioeconomic position [6]. If the advice “sit less–move more” is based on evidence predominantly from adults in high-wage, high-status, sedentary occupations (i.e., “the privileged”)—but not so from adults in low-wage, low status, physically active occupations (i.e., “less privileged”), then universal adherence to this advice could result in widening health inequalities [12]. Thus, for promoting public health and decreasing health inequalities, we see a great need for empirical testing of the proposed “Sweet-Spot Hypothesis.”

In this paper, we aim to describe the origin and theoretical underpinning of the “Sweet-Spot Hypothesis,” propose approaches to test the hypothesis, and discuss its practical implications. Our aim is to start a dialogue in the field and encourage researchers to test the hypothesis in their research. Through discussion, debates, and empirical research, we hope to expand our collective knowledge about the healthy “24-h physical behavior balance” for all.

## Origin of the “Sweet-Spot Hypothesis”

Our “Sweet-Spot Hypothesis” of 24-h physical behaviors is inspired by the pioneering work of Morris and Paffenbarger on physical behavior and health [13, 14]. Both started investigating the health effects of various physical behaviors among adults in different occupations (bus drivers, conductors and longshoremen). The selection of these “less privileged” occupations reflects the integration of occupational health and physical activity research in the early days of physical activity epidemiology [15]. Today, decades after Morris and Paffenbarger, we believe that research on physical behaviors and health in adults with different occupations can provide valuable evidence complementary to the current literature on the general adult population.

We see two main arguments for why the “Sweet-Spot” of 24-h physical behaviors of adults in physically active occupations would hypothetically differ from that of adults in sedentary occupations. Firstly, adults in physically active occupations often have physically demanding work tasks for prolonged hours for several consecutive days [6, 11, 16]. Thus, their physical behaviors at work strongly deviate from adults in sedentary occupations. Secondly, physically active occupations are often characterized by lower level of control and flexibility, putting strong constraints on the individual's physical behaviors at work [17].

The research literature underlying the current physical activity and sedentary guidelines is predominantly based on physical behaviors during leisure-time, often with overrepresentation of more privileged adults in high-status, high-wage sedentary occupations [10]. Consequently, the corpus of evidence may not be representative of the “less privileged,” and thus, the advice “sit less–move more” may not be applicable to adults in physically active occupations. For example, should we give the advice “sit less–move more” to manufacturing workers who stand for 6–7 h per day at a production line, or cleaners who spend 7–8 h a day on their feet at work? Or should we instead advise them to “sit more–move less” after work for recovery? In both cases, the advice given to sedentary office workers may not be suitable, but we do not yet have a sufficient evidence base for offering advice to these workers.

## Theoretical Underpinnings of the “Sweet-Spot Hypothesis”

The “Sweet-Spot Hypothesis” builds on the biological theory of allostasis [18], which means to “achieve stability through change.” The human body requires internal stability (homeostasis) for preserving good health during environmental changes and daily tasks and challenges. The allostatic response enables healthy adaptations by activation of various physiological systems (autonomic, cardiovascular, metabolic, inflammatory, and central) [19]. This adaptive response is for example useful for rapid energy mobilization (e.g., during a bout of MVPA) and leads to healthy habituation when the challenge is short lasting and followed by recovery (e.g., during rest). However, repeated or persistent activation or inactivation of the allostatic systems without required recovery or interruption may lead to imbalanced physiological regulation, such as altered metabolism, hypertension, or excess inflammation [20]. Such unhealthy adaptations are called allostatic load [19], which has been linked with increased risk for various diseases [21].

From a daily physical behavior and health perspective, the “Sweet-Spot” occurs when there is a balance between physical activity, sedentary behavior and sleep, leading to health-promoting allostatic adaptations. To the contrary, an imbalance between these behaviors leads to allostatic adaptations detrimental to health. For example, a long-term imbalance between extensive sedentary behavior and no MVPA may lead to unhealthy allostatic adaptations, which can compromise cardiometabolic health [22, 23]. On the contrary, a healthy 24-h physical behavior balance, with at least 30 min of MVPA per day, and sufficient, but not excessive, sedentary behavior and sleep may lead to healthy allostatic adaptations (sweet-spot), resulting in improved cardiometabolic health [24]. However, 24-h physical behavior imbalance may also occur with too much physical activity (e.g., MVPA) without sufficient sedentary time and sleep (recovery), leading to poorer cardiometabolic health [25–27]. Thus, the biological theory of allostasis makes a suitable theoretical construct for the proposed “Sweet-Spot Hypothesis.”

## How to Empirically Test the “Sweet-Spot Hypothesis”?

The “Sweet-Spot Hypothesis” postulates that the best balance between 24-h physical behaviors for better health differs between adults in sedentary and physically active occupations. Specifically, testing this hypothesis is about falsifying the null hypothesis (H_0_) that (1) there is no difference in the “Sweet-Spot” of 24-h physical behaviors between adults in occupations with different physical behaviors, and (2) the advice “sit less–move more” brings all adults from different occupations toward their “Sweet-Spot” of 24-h physical behaviors for better health.

We suggest the following steps to test the “Sweet-Spot Hypothesis.” *Firstly*, socioeconomic position is closely linked to both 24-h physical behaviors and health, and thus, it is important to sufficiently account for socioeconomic confounding. One way to do so is through study design, by ensuring study populations with considerable variance in physical behaviors at work but with a homogenous socioeconomic position. In any case, we encourage researchers to collect information on socioeconomic position (e.g., education, income and occupation).

*Secondly*, considering that self-reported measures of daily physical behaviors are susceptible to misclassification bias [28], we recommend using device-based measurements of 24-h physical behaviors. Moreover, we recommend supplementing device-based measures with self-reports or other means to ascertain the context of the physical behaviors (e.g., work, recreational, transport and domestic), as well as other conditions of the work behaviors including level of control over work tasks and amount of lifting/loading. Other important aspects of 24-h physical behaviors to consider may include day-to-day variability in behaviors (is there a consistent routine?); the timing of activities (are the most physically demanding tasks early in the morning or later in the afternoon); bout distribution (e.g., is sitting time interspersed with active breaks? Is sleep only nocturnal, or is there napping during the day); and the quality of behaviors (e.g., is MVPA from lifting heavy objects or running? Is sleep regularly disturbed?). Additionally, information on potential confounders, such as other lifestyle behaviors, physical and mental health status should be collected.

*Thirdly*, it is important to acknowledge that daily physical behaviors are parts of a finite whole where increasing time spent in one behavior necessarily means less time for other behaviors. Therefore, data on time spent in physical behaviors are compositional in nature, conveying *relative*—rather than *absolute*—information [29]. Accordingly, assessment, reporting of results and interpretation of the association between physical behaviors and health should be in relative terms, considering the co-dependency of daily physical behaviors [30, 31]. We recommend the use of analytical methods that address the nature of compositional data, such as compositional data analysis (CoDA), for analyzing relationships between times spent in physical behaviors and health [32, 33].

### An Example of Testing the “Sweet-Spot Hypothesis”

**Fig. 1:**
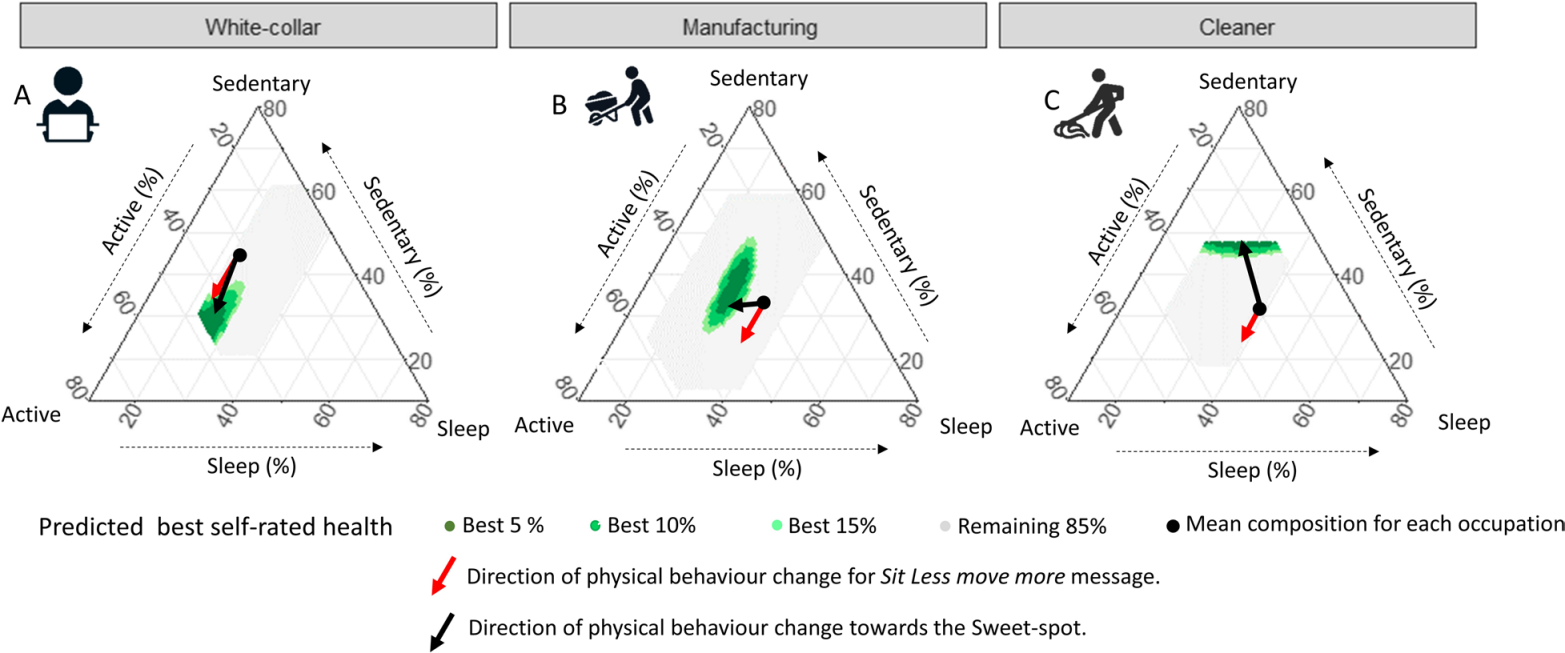
A cross-sectional association between 24-h compositional physical behaviors and self-rated health among 136 white-collar workers (**A**), 481 manufacturing workers (**B**) and 130 cleaners (**C**). For each occupation, we predicted the daily time-use composition of physical behaviors associated with the best 5% (defined as the “Sweet-Spot,” illustrated by dark green area) self-rated health, 5–10% (green area) and 10–15% (light green area) self-rated health. The gray colored area illustrates the 24-h distribution of physical behaviors for the adults we have data on. The black dot illustrates the mean composition of each occupation. The red arrow indicates the direction of physical behavioral change concordant with the advice “sit less–move more.” The black arrow indicates the direction of physical behavioral change toward the “Sweet-Spot” for better health

Figure 1 is a ternary diagram illustrating the cross-sectional association between 24-h compositional physical behaviors and self-rated health of adults in predominantly desk-based occupations (“white-collar,” Fig. 1A), manufacturing occupations (Fig. 1B) and cleaning occupations (Fig. 1C) from the DPhacto cohort [34]. We used 24-h thigh-worn accelerometry and the Acti4 software [35] to estimate the daily time spent sedentary (lying and sitting), active (standing, walking, running, cycling and stair climbing) and “in bed” (as proxy for sleep based on participants’ diary information). Following a CoDA approach, we tested the interaction between physical behaviors (transformed to isometric log-ratios) and occupation against self-rated health, using a second-order polynomial model [36]. The resulting interaction tended to be significant (*P* = 0.06), indicating differences between occupations in the association between 24-h compositional physical behaviors and self-rated health. To understand these differences, using the model estimates, we predicted the compositions of 24-h physical behaviors associated with the best 5% (defined as the “Sweet-Spot,” illustrated by the dark green areas in Fig. 1), 5–10%, and 10–15% of self-rated health within each occupation, adjusted for age, sex, body mass index and smoking.

Figure 1 visualizes the sweet-spot (green-colored areas) for adults in different occupations and shows how this “Sweet-Spot” compares to each occupation’s average daily 24-h physical behavior composition (black dot). The black arrows show the direction of physical behavior change required to bring each occupation closer toward their “Sweet-Spot.” The red arrows show the direction of physical behavior change recommended by the simple ‘move more-sit less’ recommendation.

Figure 1A shows that for adults in white-collar (administrative, mainly sedentary) occupations, the 24-h physical behavior distribution associated with the best 5% of self-rated health comprised about 30% of the day spent on sedentary behavior, 45% spent actively, and 25% spent on sleep. Therefore, the “Sweet-Spot” of 24-h physical behaviors associated with the best self-rated health appear to be achieved by following the “sit less–move more” advice, indicated by the overlapping red and black arrows from the mean composition of physical behaviors for the occupational group.

For adults working in manufacturing, Fig. 1B shows that the 24-h physical behavior distribution associated with the best 5% of self-rated health comprised about 35% spent sedentary, 35% spent actively, and 30% spent on sleep. Therefore, for adults in this occupational group, the “Sweet-Spot” of 24-h physical behaviors associated with the best self-rated health was not achieved by following the “sit less–move more” advice (indicated by the red arrow), but rather, by increasing sedentary and active time while decreasing sleep time (indicated by the black arrow).

For the adults who work in cleaning, Fig. 1C illustrates that the 24-h physical behavior distribution associated with the best 5% of self-rated health comprised about 50% spent sedentary, 15% spent actively, and 35% on sleep. For the adults in this occupation, the “Sweet-Spot” of 24-h physical behaviors was not achieved by following the advice “sit less–move more” (indicated by the red arrow), but by increasing sedentary time and decreasing time spent actively and decreasing sleep time (indicated by the black arrow).

It should be noted that this analysis merely serves as a simplified example of how to test the “Sweet-Spot Hypothesis” and it has several limitations. Firstly, given the exploratory nature, our example is based on cross-sectional analysis, which can be subject to biases. Secondly, given the somewhat small sample size, we only had statistical power to control for a few selected potential confounders. Finally, to simplify this example, we decided to combine all physical activities into one variable (i.e., “active”) but it should be acknowledged that each of these activities (i.e., standing, walking, running, cycling and stair climbing) might influence health differentially. However, the proposed approach is applicable to all study designs, and we encourage researchers to test our hypothesis in longitudinal studies, take relevant confounders into account and if possible, consider all 24-h behaviors.

## Practical Implications

We stress that the “Sweet-Spot Hypothesis” is not a contradiction to the current physical activity and sedentary behavior research and guidelines. It is not the purpose of our paper to condemn these well-developed guidelines. In fact, we applaud the physical activity and sedentary guidelines for their increasing inclusivity [37]. Instead, we propose the “Sweet-Spot Hypothesis” to encourage critical thinking, open the floor for discussions and suggest avenues for future research. By proposing the “Sweet-Spot Hypothesis,” we aim to pave the way for further development of research and more nuanced guidelines that might be better suited for particular adult population groups. Nonetheless, the specific recommendation of “sit less–move more” may be based on the assumption that the entire adult population works in a sedentary occupation, to whom physical behaviors are voluntary. However, considering that the health impact of *both* occupational and leisure-time physical behaviors among adults in physically active occupations is understudied [10], we do not yet have enough evidence to confirm whether the current recommendations could be a mismatch for this population. This gap in evidence and guidelines is what motivated us to encourage this discussion, debate and empirical research, hopefully expanding our collective knowledge about the healthy “24-h physical behavior balance” for all.

## Conclusion

In this paper, we propose the “Sweet-Spot Hypothesis.” Our main aim is to suggest a new scientific approach and to encourage research, on healthy 24-h physical behavior balance for all adults. With research on the “Sweet-Spot Hypothesis,” we see great potential in developing the evidence base required for targeted and efficient guidelines for adults across a wide range of occupations. We acknowledge that conducting such research requires interdisciplinary work across scientific fields related to physical behaviors and health [15]. Although challenging, we consider this an important next step toward developing evidence-based guidelines for all adults, and thus contributing to improving the health and equalities for all.

## Data Availability

The datasets analyzed are available at the Danish National Archives, https://www.sa.dk/en/k/about-us.

## Abbreviations

CoDA: Compositional data analysis
LMIC: Low- and middle-income countries
MVPA: Moderate-to-vigorous physical activity
WHO: The World Health Organization
